# Photo-Micography

**Published:** 1897-11

**Authors:** P. Hinman

**Affiliations:** Atlanta, Ga.


### Photo-Micography.

BY DR. P. HINMAN, ATLANTA, GA.

Abstract of paper read before the Southern Dental Society, Old Point Comfort, Va., August, 1897.

The value of photo-micography cannot be over-estimated as an illustrative method in the study of histology and pathology. The great advantage over drawings is that the mind of the artist cannot influence his pictures. In drawings, the artist whether intentionally or unintentionally, gives his views instead of the actual tissue involved. The difficulties encountered in this work are greatly over-estimated, as any one who can intelligently manage the microscope and has a limited knowledge of photography, can make photo-micographs. The apparatus can be bought very cheap. The ordinary photographic plate of any good standard is all that is necessary. Dr. Hinman then went into minute instructions in the details of the process, and exhibited a series of interesting slides of his own make, showing sections of the dentine, the alveolar process, the peridental membrane, introglobular spaces, the interzonal layer, calcific matter deposited in the root of a tooth, carious dentine, a section of enamel when Nasmythy's membrane has been worn away, exposing the ends of the enamel rods, etc., etc.

